# Evaluation of Four Indigenous Non-*Saccharomyces* Yeasts Isolated from the Shangri-La Wine Region (China) for Their Fermentation Performances and Aroma Compositions in Synthetic Grape Juice Fermentation

**DOI:** 10.3390/jof8020146

**Published:** 2022-01-30

**Authors:** Yue Zhao, Qingyang Sun, Bin Tian, Shusheng Zhu, Fei Du, Ruzhi Mao, Su Li, Lijing Liu, Yifan Zhu

**Affiliations:** 1 College of Plant Protection, Yunnan Agricultural University, Kunming 650201, China; zhaoyue_0335@126.com (Y.Z.); sunqingyang0913@163.com (Q.S.); shushengzhu79@126.com (S.Z.); dufeifei2018@163.com (F.D.); 2 College of Food Science, Hebei Normal University of Science & Technology, Qinhuangdao 066600, China; 3 Faculty of Agriculture and Life Sciences, Lincoln University, Lincoln 7647, New Zealand; bin.tian@lincoln.ac.nz; 4 College of Food Science and Technology, Yunnan Agricultural University, Kunming 650201, China; 18082970271@163.com (R.M.); cherry.liu@vip.163.com (L.L.); 5 University Engineering Research Center for Grape & Wine of Yunan Province, Yunnan Agricultural University, Kunming 650201, China; 6 College of Horticulture and Landscape, Yunnan Agricultural University, Kunming 650201, China; evillife_t@hotmail.com

**Keywords:** pure culture fermentation, *Meyerozyma guilliermondii*, *Saccharomycopsis vini*, *Saturnispora diversa*, *Wickerhamomyces anomalus*

## Abstract

This study investigated the fermentation performances and aroma compositions of synthetic grape juice that was fermented by four indigenous non-*Saccharomyces* yeast isolates that were obtained from the Shangri-La wine region (China): *Meyerozyma guilliermondii* (AD-58), *Saccharomycopsis vini* (BZL-28), *Saturnispora diversa* (BZL-11), and *Wickerhamomyces anomalus* (DR-110), in comparison to those of *Saccharomyces cerevisiae* (EC1118). The four indigenous non-*Saccharomyces* yeasts showed a lower fermentative capacity and a lower conversion rate of sugar to alcohol, but a higher yield of volatile acidity. *W. anomalus* (DR-110) had a greater ability to produce numerous esters and short-chain fatty acids and the representative flavors of its fermented medium were fruity and fatty. *Sac.* *vini* (BZL-28), interestingly, exhibited great capacity in the formation of many monoterpenes, particularly (*Z*)-β-ocimene, *E*-β-ocimene, linalool, citral, and geraniol and its fermented medium was characterized by a strong fruity (citrus-like) and floral flavor. *M. guilliermondii* (AD-58) and *Sat. diversa* (BZL-11) only mildly affected the aroma profiles of their resultant fermented media, since the concentrations of most of the volatiles that were produced by these two isolates were much lower than their sensory thresholds. The four indigenous non-*Saccharomyces* yeasts exhibited distinctive fermentation performances and aroma production behaviors. In particularly, *W. anomalus* (DR-110) and *Sac. vini* (BZL-28) have shown good potential in enhancing the aromas and complexity of wine.

## 1. Introduction

Wine fermentation is a complex biochemical process that is conducted by many different microorganisms. *Saccharomyces* and non-*Saccharomyces* yeasts are the predominant microorganisms that are responsible for wine’s fermentation [1]. In the past, non-*Saccharomyces* yeasts were usually isolated from sour wine and associated with unpleasant flavor, therefore oenologists tend to inhibit the activity of these yeasts during wine fermentation [2]. By contrast, *S. cerevisiae* yeasts are more favored in wine production for their reliable fermentation characteristics with consistent quality [2].

Recent studies have revealed the new roles of non-*Saccharomyces* yeasts in wine production, particularly for some specific enological characteristics. For example, *Hanseniaspora*, *Metschnikowia*, *Starmerella,* and *Zygosaccharomyces* have shown a lower sugar–ethanol conversion rate during fermentation, thus they could be applied to produce a reduced-alcohol wine [3,4]. Furthermore, some non-*Saccharomyces* yeasts like *Lachancea thermotolerans*, *Pichia kudriavzevii*, and *Schizosaccharomyces pombe* have shown potential in regulating acidity during wine fermentation, which could be used for improving the quality of must containing excessive or insufficient acidity [5,6,7]. Several strains of *Aureobasidium pullulans*, *Cystofilobasidium capitatum*, *Cryptococcus saitoi*, *Rhodosporidiobolus colostri*, and *Rhodotorula dairenensis* have shown great capability of improving the color quality of wines by producing pectinase during fermentation [8].

The aroma is one of the most important sensory properties of wine and the influence of non-*Saccharomyces* yeasts on the wine’s aroma has always been the main focus of wine research. Compared with *S*. *cerevisiae*, some non-*Saccharomyces* yeasts, such as *Metschnikowia pulcherrima*, *Pichia kudriavzevii*, *Torulaspora delbrueckii*, *Lachancea thermotolerans,* and *Zygosaccharomyces bailii* have exhibited unique behaviors in producing volatile aroma compounds [6,9]. Consequently, the application of non-*Saccharomyces* yeasts in the production of wine or other beverages could have a great contribution to their aromatic complexity [7,10,11,12,13]. In addition, most of the non-*Saccharomyces* yeasts are good producers of highly active glycosidase (typically β-D-glucosidase) [14,15]. These enzymes could release varietal aromas, such as terpenols, terpene diols, and C_13_-norisoprenoids from the corresponding precursors that existed in the grape must [16]. This feature of the non-*Saccharomyces* yeasts has also been employed by some researchers in order to enhance the primary aroma of wine [17,18].

Diverse non-*Saccharomyces* yeasts are widely distribute in the vineyards and on the surface of grapes [19,20]. Although some of them have been confirmed to exhibit unique performances during fermentation, there are still many undiscovered oenological behaviors of non-*Saccharomyces* yeasts that deserve to be explored. Shangri-La is a distinctive Chinese wine region that has the highest altitude vineyards and is rich in biodiversity. In our previous work, a number of non-*Saccharomyces* yeast isolates have been found in this region [21], but most of them have not been studied for their potential application in wine production.

Therefore, the aim of the present study was to explore the oenological behaviors of four indigenous non-*Saccharomyces* yeast isolates that were obtained from the Shangri-La wine region (China), including *Meyerozyma guilliermondii* (AD-58), *Saccharomycopsis vini* (BZL-28), *Saturnispora diversa* (BZL-11)*,* and *Wickerhamomyces anomalus* (DR-110). To this end, the fermentation performances of these four isolates in synthetic grape juice have been studied and compared to a commercial *Saccharomyces cerevisiae* yeast (EC1118). The aroma compositions in the resultant fermented media have also been analyzed for their potential contribution to aroma enhancement and regional characteristics.

## 2. Materials and Methods

### 2.1. Microorganisms

The four non-*Saccharomyces* yeast isolates, *Meyerozyma guilliermondii* (*M. guilliermondii*, AD-58), *Saccharomycopsis vini* (*Sac. vini*, BZL-28), *Saturnispora diversa* (*Sat. diversa*, BZL-11), and *Wickerhamomyces anomalus* (*W. anomalus*, DR-110), were obtained from the spontaneous fermentation of Cabernet Sauvignon wines in the Shangri-La wine region, Yunnan, China [21]. A commercial *Saccharomyces* *cerevisiae* yeast (*S.* *cerevisiae*, EC1118) (Lallemand Inc., Montreal, QC, Canada) was used for comparison. The colony morphologies, on YPD and WL agar, of these non-*Saccharomyces* yeast isolates have been presented in Figure S1. The detailed information of the 26S rRNA D1/D2 region and the 5.8S rRNA ITS region of these non-*Saccharomyces* yeast isolates are listed in Table S1. The phylogenetic trees of the four non-*Saccharomyces* yeast isolates, based on the sequence analyses of the 26S rRNA D1/D2 region and 5.8S rRNA ITS region, have been presented in Figures S2 and S3.

### 2.2. Fermentations in Synthetic Grape Juice

Fermentation was carried out in triplicate in 500 mL Erlenmeyer flasks containing 300 mL of synthetic grape juice (SGJ). The SGJ was prepared according to the manufacturer’s instructions (Shandong Tuopu Biol-engineering Co., Ltd., Zhaoyuan, Shandong, China) and sterilized by filtration (0.22 μm sterile membrane). The detailed compositions of the SGJ (1 L) are as follows: 90 g D-glucose, 90 g D-fructose, 3 g L-tartaric acid, 0.3 g L-malic acid, 0.3 g citric acid, 2.0 g KH_2_PO_4_, 0.2 g MgSO_4_·7H_2_O, 0.3 g (NH_4_)_2_SO_4_, 0.6 g Asparagine, 4 mg MnSO_4_·H_2_O, 4 mg ZnSO_4_·7H_2_O, 1 mg CuSO_4_·5H_2_O, 1 mg KI, 1 mg H_3_BO_3_, 1 mg (NH_4_)_6_Mo_7_O_24_·4H_2_O, 0.4 mg CoCl_2_·6H_2_O, 1 mg 4-Aminobenzoic acid, 0.3 g Inositol, 1 mg Vitamin B1 (Thiamine), 1 mg Vitamin B3 (Nicotinic acid), 1 mg Vitamin B5 (Pantothenic Acid), 1 mg Vitamin B6 (Pyridoxine), and 0.04 mg Vitamin H (Biotin). The pH was adjusted to 3.5 with KOH.

Cultures of the four non-*Saccharomyces* yeast isolates and EC1118 were pre-incubated in 10 mL of YPD medium (10 g/L yeast extract, 20 g/L peptone, and 20 g/L glucose) at 28 °C in a rotary shaker (100 rpm) for 48 h in order to get an inoculum size of 10^7^ CFU/mL. Then, the SGJ was inoculated with 1% (*v*/*v*) of the pre-cultures and incubated at 28 °C (100 rpm) for fermentation. The flasks were sealed with 8 layers of sterile gauze in order to allow the release of CO_2_ and also to prevent external microbial contamination. The fermentation processes of the four non-*Saccharomyces* yeast isolates and EC1118 were monitored daily by measuring the weight loss until the end of the fermentation (less than 0.10 g of weight loss for three consecutive days).

### 2.3. Oenological Parameters Analysis

The oenological parameters including the reducing (residual) sugar concentration, alcohol content, pH, total acidity concentration (expressed as tartaric acid), and volatile acidity concentration (expressed as acetic acid) were measured according to the National Standard of the People’s Republic of China: GB/T 15038-2006, *Analytical Methods of Wine and Fruit Wine*. The concentration of reducing sugar was determined by using the 3,5-dinitrosalicylic acid method. The content of alcohol was determined by utilizing the density method. The concentration of the total acidity was determined by titration using standard sodium hydroxide (0.05 M). The separation of volatile acidity from the wine was carried out by steam distillation and the concentration of volatile acidity was titrated by standard sodium hydroxide (0.05 M). The value of pH was determined by a pH meter.

### 2.4. Volatile Aroma Compounds Analysis

The volatile aroma compounds in the five fermented media and SGJ were determined using the method that was reported by Zhang [22], with some modifications.

The volatiles were extracted by using the headspace solid phase microextraction (HS-SPME) method. Briefly, the fermented medium (5 mL), NaCl (1.0 g), and internal standard (4-methyl-2-pentanol, with a final concentration of 2030 μg/L) were blended in a 15 mL airtight vial with PTFE/Silicone septa (27159, Supelco, Bellefonte, PA, USA) containing a magnetic stirrer. After being equilibrated at 40 °C for 30 min under a rotational speed of 250 rpm, the sample was extracted by an SPME manual device (57330-U, Supelco, Bellefonte, PA, USA) that was equipped with DVB/CAR/PDMS fiber (57328-U, Supelco, Bellefonte, PA, USA) for 30 min with continued heating and rotation. Afterward, fiber was inserted into the GC injection port to desorb for 10 min at 250 °C (splitless model) and begin the GC-MS analysis.

The separation and identification of the volatiles were achieved in an Agilent 7890B GC system that was coupled with an Agilent 5977A MS detector and equipped with a DB-Wax capillary column (60 m × 0.250 mm i.d., 0.25 μm df, J&W scientific, Agilent, Santa Clara, CA, USA). Ultra-pure helium (99.999%) was used as a carrier gas at a flow rate of 1 mL/min. The temperature program that was used was as follows: the process started at 50 °C for 1 min and then increased to 220 °C at 3 °C/min with a final holding time of 5 min; the total run time was 62.7 min. The temperature of the injector, transfer line, and ion source was set to 250 °C. The mass spectra were obtained using a mass selective detector (MSD) working in electronic impact at 70 eV in scan mode with a mass range of 30–350 *m*/*z*.

The identification of the volatiles was done by comparing the retention indices (RIs) and mass spectra with those of the pure standards as sourced from the NIST Chemistry WebBook (https://webbook.nist.gov/, accessed on 2 December 2021) and the standard NIST 14 library. The RIs were calculated using the C_10_-C_24_ alkane standard mixture (all even, soluble in heptane) (Sigma, city, Switzerland) under the same chromatographic conditions as the fermented media.

The quantitative analysis was performed using the internal standard–standard curve method; 4-methyl-2-pentanol was used as the internal standard and the standard curve was plotted using the 5-point method. According to the alcohol content, total acidity concentration, and pH in the fermented media, two kinds of synthetic model wine solution (1.0% and 8.0% *v*/*v* alcohol content, 5.0 g/L tartaric acid, and adjusted pH to 3.4 with KOH) were prepared. All of the pure standards were dissolved in ethanol together and then this mixed solution was diluted to different levels with the synthetic model wine solutions. The mixed standards of each level were extracted and analyzed under the same conditions as the fermented media. When a volatile standard was a mixture of two isomers (e.g., β-Ocimene), the total areas of these two isomers were employed to plot the standard curve. When plotting a volatile compound for which there was no pure standard, the concentration of it was estimated by the standard curve of the standard compound with the most similar chemical structure or expressed as relative amount compared to the internal standard. The quantitative standard curves of each compound are listed in Table S2.

### 2.5. Odor Activity Values (OAVs) and Aroma Series Analysis

The odor activity values (OAVs), a commonly used parameter for the evaluation of the contribution of volatiles to wine aroma [23,24,25,26], were calculated and expressed as the ratio between the concentration of an individual compound and its perception threshold.

To predict the overall aroma profile of the fermented media from the GC-MS analysis data, the aroma-active compounds were grouped into six aroma series based on similar odor descriptions. These six aroma series were modeled from the literature [23,25,26,27,28,29,30], they included fruity, floral, herbaceous (green), balsamic, solvent, and fatty.

Due to the high complexity of olfactory descriptions, some aroma-active compounds might be included in two or more aroma series [25,29,30]. The total intensities for each aroma series were calculated by accumulating the OAVs of the individual compounds that belonged to each series, as listed in Table 3 (the compounds with OAVs > 1.0).

### 2.6. Statistical Analysis

All of the data were subjected to a one-way analysis of variance (ANOVA) that was performed through the IBM SPSS statistics 19.0 software package (SPSS Inc., Chicago, IL, USA.) employing Duncan multiple range tests at a significance level of *p* < 0.05. The results were expressed as the mean value ± the standard deviation. The principal component analysis (PCA) was performed through Origin 2018 (OriginLab Corporation, Northampton, MA, USA).

## 3. Results and Discussion

### 3.1. Fermentation Kinetics and Oenological Parameters Analysis

**Figure 1:**
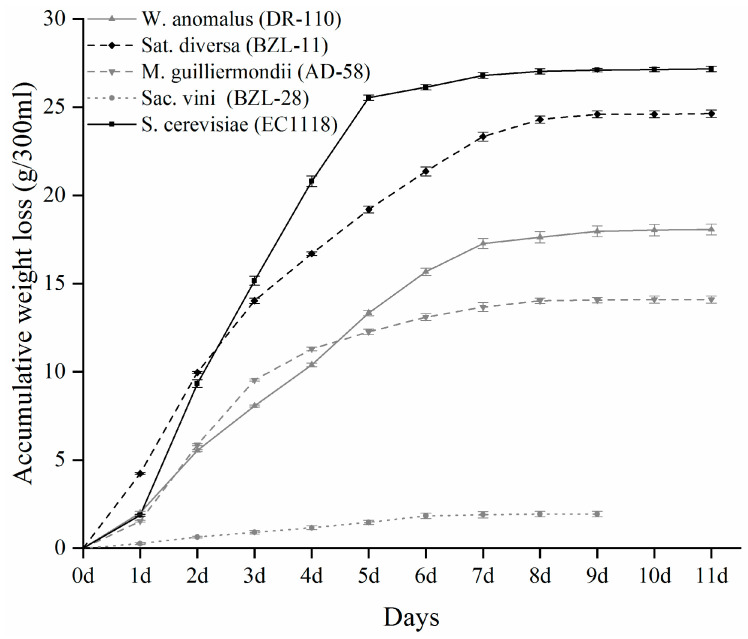
Fermentation kinetics of four indigenous non-*Saccharomyces* yeast isolates and EC1118 in SGJ.

The fermentation kinetics of the five yeasts and the oenological parameters of the resultant fermented media are shown in Figure 1 and Table 1, respectively. The four non-*Saccharomyces* yeast isolates exhibited lower fermentative capacity when compared with the *S*. *cerevisiae* yeast EC1118. This observation was consistent with those of many other non-*Saccharomyces* yeasts that have been reported in previous studies [31,32]. *Sat. diversa* (BZL-11) showed the highest fermentation capacity among the four non-*Saccharomyces* yeast isolates, with 8.41% abv produced from 90.6% reducing sugars in SGJ within 9 days. In contrast, *Sac. vini* (BZL-28) barely started the fermentation with only 0.06% abv produced at the end of fermentation. *M. guilliermondii* (AD-58) and *W. anomalus* (DR-110) exhibited an intermediate fermentative behavior. In addition, these non-*Saccharomyces* yeast isolates showed a lower rate of conversion of sugar to alcohol (Table 1), which indicated that they might be potentially used for low-alcohol wine production [3,4].Compared with that which was fermented by EC1118 (0.25 g/L), a higher level of volatile acidity was observed in the media that was fermented by the four non-*Saccharomyces* yeast isolates (ranging between 0.44 and 1.97 g/L), particularly in those that were fermented by *M. guilliermondii* (AD-58) and *W. anomalus* (DR-110). This may also explain the higher concentration of the total acidity in these two fermented media. As reported previously, the excessive production of volatile acidity is the main problem for most of the non-*Saccharomyces* yeasts [10,31,33]. Co-inoculation of non-*Saccharomyces* yeasts with *S. cerevisiae* [3,7] and reducing the oxygen saturation during fermentation [34] could be used to reduce the yield of volatile acidity from non-*Saccharomyces* yeasts.

### 3.2. Volatile Aroma Compounds Analysis

A total of 62 volatile aroma compounds were detected in the four non-*Saccharomyces* yeast isolates and EC1118 fermented media and the SGJ by HS-SPME-GC-MS. These volatile aroma compounds can be classified into six groups, including alcohols (16), esters (21), terpenes (10), fatty acids (5), carbonyl (8), and others (2). The qualitative and quantitative information of these aroma compounds are listed in Table 2. The odor activity values (OAVs) of 22 key aroma compounds (with OAVs > 1.0) in the five fermented media and SGJ are listed in Table 3. As shown in Table 2, very few volatile aroma compounds were detected in the SGJ. This result indicated that the aroma production characteristics of the four indigenous non-*Saccharomyces* yeast isolates could be objectively evaluated by the SGJ fermentation, since the aroma compositions of their fermented media were not affected by the varietal aromas that derived from the natural grape juice.

ALCOHOLS. Higher alcohols (aliphatic and aromatic alcohols) are by-products of yeast metabolism during alcoholic fermentation. These compounds could add desirable complexity to the wine’s aroma at a low concentration (below 300,000 μg/L), whereas they may have a detrimental effect when their concentration exceeds 400,000 μg/L [35]. In the present work, the commercial *S. cerevisiae* yeast (EC1118) produced the highest total concentration of higher alcohols during the fermentation (358,053.00 μg/L), a result which was also observed in previous studies [7,32]. Whereas the total concentrations of higher alcohols that were generated by the four non-*Saccharomyces* yeast isolates were all below 300,000 μg/L (ranging between 6833.32 and 221,829.74 μg/L), suggesting that these non-*Saccharomyces* yeast isolates could positively enhance the complexity of wine aroma. The major alcohols that were detected in this study were 1-propanol, 2-methyl-1-propanol, 3-methyl-1-butanol, 3-ethoxy-1-propanol, 3-methylthio-1-propanol, and 2-phenylethanol. Most of the major higher alcohols were detected at a higher level in the fermented media of *Sat.* *diversa* (BZL-11) and *W. anomalus* (DR-110). Of those higher alcohols, the concentration of 2-phenylethanol (63,510.12 μg/L) was determined to be present at a significantly higher level in the fermented medium of *Sat.* *diversa* (BZL-11). The lowest concentration of higher alcohols was observed in the fermented medium of *Sac. vini* (BZL-28) due to its weak fermentative capability (Table 2).

The 2,3-butanediol represents the most abundant volatile by-product of alcoholic fermentation and can affect both the bouquet of the wine due to its bitter taste and the body of the wine due to its viscosity [36]. In the present study, two isomers of 2,3-butanediol (the R,R- and R,S-form) were detected (except for in the medium that was fermented by *Sac. vini*). As shown in Table 2, the *S. cerevisiae* yeast (EC1118) exhibited a strong capacity for 2,3-butanediol production (576,699.62 and 122,089.94 μg/L for 2R,3R-butanediol and 2R,3S-butanediol, respectively), which was in agreement with the results that were reported by Romano [36]. *W. anomalus* (DR-110) showed a similar capability of 2,3-butanediol production as EC1118 with regard to the ratio of the two isomers and the total concentration of 2,3-butanediol (Table 2). *M. guilliermondii* (AD-58) also produced a high level of 2,3-butanediol during fermentation with more than 90% of the R,R-form of 2,3-butanediol (537,151.25 μg/L). These two isomers of 2,3-butanediol were observed at a relatively low level in the fermented medium of *Sat. diversa* (BZL-11), despite the fact that this yeast exhibited a greater alcoholic fermentation capability. On must take into account that the fermented medium of *Sat. diversa* (BZL-11) contained a relatively high level of acetoin (25,524.16 μg/L), the precursor of 2,3-butanediol, which could be explained by the relatively low activity of the acetoin reductase in *Sat. diversa* (BZL-11).

EASTER. Esters have long been regarded as important contributors to wine aroma because they are the primary source of fruity aromas [37]. In the present study, a total of 21 esters were detected in the fermented media of the four non-*Saccharomyces* yeast isolates and EC1118. As shown in Table 2, ethyl acetate was the most abundant ester in the five fermented media. The highest concentration of this compound (143,023.77μg/L) was observed in the medium that was fermented by *W. anomalus* (DR-110), which was approximately 7-fold higher than that which was found in the medium that was fermented by EC1118 (19,384.40 μg/L). Additionally, *W. anomalus* (DR-110) produced the most abundant esters during fermentation and some of these esters were exclusively found in its fermented medium, notably fatty acid esters of higher alcohols (Table 2). However, these compounds were not detected in apple cider that was fermented by *W. anomalus* (YN6) [38]. This could be explained by the difference of the ester metabolisms among different *W. anomalus* strains or the difference of the components between apple juice and SGJ. Therefore, the distinctive ester production ability of *W. anomalus* (DR-110) requires further study. Acetate esters and fatty acid ethyl esters were the primary esters that were detected in the fermented medium of EC1118, which is in agreement with previous studies [32,39]. The esters that were identified in the fermented medium of *Sat. diversa* (BZL-11) were similar to those of EC1118 but with a much lower concentration (Table 2). The exclusive presence of geranyl acetate in the fermented medium of *Sac.* *Vini* (BZL-28) could be related to the high production of geraniol of this isolate (Table 2). Concomitantly, according to the data that were obtained in the present study, *M. guilliermondii* (AD-58) seems not to be good at producing esters during fermentation.

TERPENES. Terpenes are the typical aroma compounds contributing to fruity (citric-like) and floral characters in wine [35] and they can exist as free and glycosylated precursors in grapes [40]. Although the SGJ that was used in the present work did not contain any free or glycosylated precursors of terpenes, several monoterpenes were still detected in the resultant fermented media of four of the yeasts, including *M. guilliermondii* (AD-58), *Sat. diversa* (BZL-11), *Sac.* *vini* (BZL-28), and *S. cerevisiae* (EC1118). This result was in agreement with previous studies which revealed that some *S. cerevisiae* and non-*Saccharomyces* yeasts have the ability of de novo biosynthesis of monoterpenes [40,41,42]. The concentrations of monoterpenes were determined at a trace level in the fermented media of *M. guilliermondii* (AD-58), *Sat. diversa* (BZL-11), and *S. cerevisiae* (EC1118) (Table 3), which is also in agreement with the results of previous studies [41,42]. In this study, *Sac.* *vini* (BZL-28) exhibited a notably high yield of terpenes during fermentation with more than ten monoterpenes having been detected in its fermented medium. Geraniol was the principal monoterpene, with the highest concentration at 1936.43 μg/L, that was observed in the medium that was fermented by *Sac.* *vini* (BZL-28), which was nearly 65-fold higher than its sensory threshold of 30 μg/L. In addition, (*Z*)-β-ocimene, (*E*)*-*β-ocimene, linalool, and citral also showed high OAVs in the medium that was fermented by *Sac. vini* (BZL-28) (Table 3). These results indicate that *Sac.* *vini* (BZL-28) could be used for improving the aroma of wines that are produced from neutral or low-aromatic grape varieties.

VOLATILE FATTY ACIDS. Volatile fatty acids are normally described as fatty, rancid, or cheesy odors (Table 2). They could contribute to the complexity of a wine’s aroma at a low concentration under their sensory thresholds [35]. Among the five volatile fatty acids that were determined in the present work, short-chain fatty acids (isobutyric acid, butanoic acid, and isovaleric acid) were mainly detected in the fermented media of the non-*Saccharomyces* yeast isolates, with the highest concentration of short-chain fatty acids having been observed in the fermented medium of *W. anomalus* (DR-110) (21,679.20 μg/L). Medium-chain fatty acids, including octanoic acid and decanoic acid, were mainly detected in the fermented medium of EC1118. This result was consistent with a previous study that found that cherry wine that was fermented by *S. cerevisiae* yeasts (EC1118 and D254) showed higher levels of medium-chain fatty acids than those that were fermented by non-*Saccharomyces* yeasts (e.g., *T. delbrueckii* and *M. pulcherrima*) [32]. The ability of *S. cerevisiae* yeasts to produce medium-chain fatty acids may enhance their competitiveness during wine fermentation, since medium-chain fatty acids could inhibit the growth of some non-*Saccharomyces* yeasts and bacteria [43,44,45].

CARBONYL AND OTHER COMPOUNDS. In this study, two aldehydes (nonanal and benzaldehyde), six ketones (methyl isobutyl ketone, 3-penten-2-one, 5-methyl-2-hexanone, acetoin, 6-methyl-5-hepten-2-one, and 2-nonanone), 1-(1-ethoxyethoxy)-pentane, and *γ*-butyrolactone were determined in the fermented media of the four non-*Saccharomyces* yeast isolates and EC1118 (Table 2). Although differences in their concentrations were observed between the five fermented media, only the concentration of nonanal was determined at the level near to, or beyond, its sensory threshold of 15 μg/L. As nonanal was also observed in the uninoculated SGJ, this aroma compound seems unlikely to be associated with the fermentation of these yeasts.

**Table 2:** Concentrations of volatile aroma compounds in five fermented media and SGJ (mean ± SD).

No.	RI ^a^	Compounds ^b^	Concentrations (μg/L)						Odor Threshold (μg/L) ^c^	Odor Description ^d^
			*M. guilliermondii* (AD-58)	*Sat. diversa* (BZL-11)	*Sac. vini* (BZL-28)	*W. anomalus* (DR-110)	*S. cerevisiae* (EC1118)	SGJ (Uninoculated)		
1	1036	1-Propanol ^A^	8408.27 ± 197.62 ^c^	2284.48 ± 57.42 ^d^	759.35 ± 34.64 ^d^	18,813.98 ± 1067.56 ^b^	42,118.28 ± 2014.65 ^a^	nd	306,000 [29,30]	Alcohol, ripe fruit [30]
2	1085	2-Methyl-1-propanol ^A^	16,932.19 ± 561.49 ^d^	26,174.41 ± 675.13 ^c^	tr	31,727.84 ± 1142.44 ^b^	37,657.46 ± 2447.08 ^a^	nd	40,000 [46]	Alcohol, solvent [30]
3	1142	Butanol ^A^	nd	nd	nd	168.30 ± 9.33	nd	nd	150,000 [29,30]	Medicinal, phenolic [30]
4	1213	3-Methyl-1-butanol ^A^	21,680.11 ± 530.17 ^c^	129,185.54 ± 8171.46 ^b^	5572.99 ± 496.70 ^c^	130,776.34 ± 2122.26 ^b^	250,240.90 ± 13,174.17 ^a^	nd	30,000 [46]	Alcohol, nail polish [30]
5	1254	3-Methyl-3-buten-1-ol ^A^	25.44 ± 3.09 ^b^	25.42 ± 4.88 ^b^	nd	50.95 ± 5.72 ^a^	nd	nd	600 [24]	Alcohol, solvent *
6	1333	3-Methyl-1-pentanol ^A^	nd	32.15 ± 3.63 ^b^	nd	22.03 ± 0.54 ^c^	62.44 ± 3.57 ^a^	nd	1000 [29,30]	Green, solvent [30]
7	1386	3-Ethoxy-1-propanol ^A^	tr	nd	nd	tr	528.73 ± 20.45	nd	100 [29,30]	Fruity [30]
8	1498	2-Ethyl-1-hexanol ^A^	5.80 ± 0.12 ^b^	8.41 ± 0.93 ^a^	2.16 ± 0.07 ^c^	5.95 ± 0.03 ^b^	4.82 ± 0.07 ^bc^	2.33 ± 0.09	8000 [24]	Waxy, soapy *
9	1527	2-Nonanol ^C^	nd	nd	8.01 ± 0.24 ^b^	nd	11.52 ± 0.27 ^a^	nd	NF	Green [26]
10	1566	Octanol ^A^	nd	nd	nd	5.62 ± 0.15 ^a^	5.31 ± 0.25 ^a^	4.37 ± 0.03	800 [29,30]	Lemon, jasmine [30]
11	1669	Nonanol ^A^	7.04 ± 0.05 ^b^	nd	7.42 ± 0.10 ^a^	7.52 ± 0.05 ^a^	nd	nd	600 [24]	Fruity, sweet [26]
12	1730	3-Methylthio-1-propanol ^A^	nd	472.82 ± 57.90 ^b^	nd	497.08 ± 15.85 ^b^	730.88 ± 14.66 ^a^	nd	1000 [47]	Cooked potato, garlic [30]
13	1891	Benzyl alcohol ^A^	tr	136.39 ± 12.53 ^b^	240.85 ± 24.83 ^a^	nd	nd	nd	900,000 [29,30]	Toasted [30]
14	1928	2-Phenylethanol ^A^	3743.17 ± 137.52 ^d^	63,510.12 ± 2979.73 ^a^	242.53 ± 51.51 ^e^	17,405.83 ± 404.72 ^c^	26,692.65 ± 439.28 ^b^	nd	10,000 [46]	Roses [30]
		∑ Higher alcohols	50,802.02 ± 1261.85 ^d^	221,829.74 ± 11,647.04 ^b^	6833.32 ± 583.81 ^e^	199,481.44 ± 3552.16 ^c^	358,053.00 ± 13,985.63 ^a^	6.70 ± 0.12		
1	1549	2R,3R-Butanediol ^C^	537,151.25 ± 44,873.06 ^b^	231,750.33 ± 12,660.86 ^c^	nd	622,811.33 ± 29,652.85 ^a^	576,699.62 ± 24,216.94 ^ab^	nd	150,000 [29,30]	Fruity [30]
2	1585	2R,3S-Butanediol ^A^	55,984.92 ± 4112.28 ^b^	25,422.53 ± 839.23 ^c^	nd	130,128.37 ± 7072.80 ^a^	122,089.94 ± 4230.99 ^a^	nd	150,000 [29,30]	Fruity [30]
		∑ Polyols	593,136.16 ± 48,953.50 ^b^	257,172.86 ± 13,477.34 ^c^		752,939.70 ± 36590.38 ^a^	698,789.56 ± 26,073.88 ^a^			
1	933	Ethyl acetate ^A^	6706.40 ± 319.00 ^d^	1735.61 ± 234.45 ^d^	74,106.81 ± 2945.79 ^b^	143,023.77 ± 4894.98 ^a^	19,384.40 ± 886.41 ^c^	tr	7500 [46]	Pineapple, varnish, balsamic [30]
2	985	Propyl acetate ^A^	tr	nd	13.05 ± 0.88 ^b^	108.02 ± 2.57 ^a^	nd	nd	4700 [29,30]	Celery [29]
3	1118	3-Methylbutyl acetate ^A^	50.89 ± 3.62 ^c^	14.22 ± 3.52 ^c^	106.22 ± 27.76 ^c^	507.00 ± 25.80 ^a^	376.16 ± 22.71 ^b^	nd	30 [46]	Fruity, sweet [30]
4	1764	Geranyl acetate ^A^	nd	nd	620.86 ± 42.94	nd	nd	nd	NF	Roses, lavender *
5	1829	2-Phenylethyl acetate ^A^	17.54 ± 0.35 ^d^	68.34 ± 4.10 ^b^	nd	42.13 ± 2.73 ^c^	135.35 ± 4.60 ^a^	nd	250 [46]	Fruity [30]
		∑ Acetate esters	6774.82 ± 315.20 ^d^	1818.17 ± 233.44 ^e^	74,846.94 ± 2948.03 ^b^	143,680.92 ± 4871.63 ^a^	19,895.90 ± 877.40 ^c^			
1	974	Ethyl propaonate ^A^	nd	nd	tr	280.82 ± 23.68 ^b^	352.76 ± 19.68 ^a^	nd	1800 [29,30]	Apple, banana [30]
2	979	Ethyl 2-methylpropanoate ^A^	nd	nd	nd	318.47 ± 69.69	nd	nd	15 [47]	Fruity [30]
3	1048	Ethyl 2-methylbutyrate ^A^	nd	nd	nd	126.49 ± 12.26	nd	nd	18 [47]	Fruity [26]
4	1063	Ethyl 3-methylbutyrate ^A^	nd	nd	nd	4.37 ± 1.17	nd	nd	3 [47]	Fruity [26]
5	1239	Ethyl hexanoate ^A^	nd	12.05 ± 0.51 ^b^	nd	10.99 ± 0.35 ^b^	435.23 ± 49.59 ^a^	nd	5 [46]	Green apple, banana [30]
6	1444	Ethyl octanoate ^A^	nd	30.89 ± 4.34 ^b^	nd	nd	217.02 ± 8.37 ^a^	nd	2 [46]	Fruity, sweet [30]
7	1649	Ethyl decanoate ^A^	nd	37.03 ± 3.82 ^b^	nd	nd	142.44 ± 14.40 ^a^	nd	200 [47]	Fruity, rose, waxy [26]
8	1797	Ethyl phenylacetate ^A^	nd	24.80 ± 1.02 ^a^	nd	17.69 ± 0.13 ^c^	22.38 ± 0.54 ^b^	nd	73 [48,49]	Honey *
9	1854	Ethyl dodecanoate ^A^	19.14 ± 0.11 ^c^	22.01 ± 1.09 ^b^	nd	19.41 ± 0.16 ^c^	36.95 ± 1.30 ^a^	nd	1500 [24]	Fruity, floral, sweet, cream [26]
10	2053	Ethyl tetradecanoate ^D^	9.58 ± 1.40 ^b^	37.07 ± 1.82 ^a^	nd	41.89 ± 3.66 ^a^	nd	nd	2000 [24]	Mild waxy, soapy [26]
11	2243	Ethyl hexadecanoate ^A^	nd	51.60 ± 3.00	nd	nd	nd	nd	1500 [24]	Fruity, sweet, fatty [26]
		∑ Fatty acid ethyl esters	28.72 ± 1.46 ^d^	215.46 ± 8.03 ^c^		883.13 ± 95.74 ^b^	1206.77 ± 30.13 ^a^			
1	1182	2-Methylpropyl 2-methylbutanoate ^D^	nd	nd	nd	31.68 ± 6.59	nd	nd	NF	NF
2	1196	3-Methylbutyl propionate ^A^	nd	nd	nd	8.88 ± 0.58 ^b^	10.72 ± 0.29 ^a^	nd	NF	Fruity *
3	1202	3-Methylbutyl 2-methylpropanoate ^D^	nd	nd	nd	176.61 ± 20.70	nd	nd	NF	NF
4	1285	3-Methylbutyl 2-methylbutanoate ^D^	nd	nd	nd	28.42 ± 5.80	nd	nd	NF	NF
5	1288	2-Methylbutyl 2-methylbutanoate ^D^	nd	nd	nd	15.39 ± 3.97	nd	nd	NF	NF
		∑ other esters				260.98 ± 24.34 ^a^	10.72 ± 0.29 ^b^			
1	1163	β-Myrcene ^A^	nd	nd	68.38 ± 6.19	nd	nd	nd	100 [49]	Lemon, pine *
2	1207	D-Limonene ^A^	nd	nd	22.70 ± 1.33	nd	nd	nd	200 [49]	Citrus, floral, green [28]
3	1238	(*Z*)-β-Ocimene ^B^	nd	nd	111.25 ± 10.62	nd	nd	nd	34 [49]	Fruity [28]
4	1256	(*E*)-β-Ocimene ^B^	nd	nd	175.79 ± 18.76	nd	nd	nd	34 [49]	Fruity [28]
5	1554	Linalool ^A^	nd	nd	28.75 ± 2.09	nd	nd	nd	15 [46]	Citrus, floral [30]
6	1711	*α*-Terpineol ^A^	10.20 ± 0.01 ^b^	10.28 ± 0.20 ^b^	9.50 ± 0.02 ^a^	nd	nd	nd	250 [47]	Floral [26]
7	1745	Citral ^A^	nd	nd	375.83 ± 28.54	nd	nd	nd	85.3 [50]	Citrus *
8	1774	Citronellol ^A^	10.47 ± 0.29 ^b^	nd	87.28 ± 3.96 ^a^	nd	17.89 ± 0.58 ^b^	nd	100 [46]	Rose [30]
9	1810	Nerol ^A^	15.74 ± 0.10 ^b^	15.89 ± 0.03 ^b^	33.30 ± 2.71 ^a^	nd	nd	nd	700 [48,51]	Floral [25]
10	1858	Geraniol ^A^	19.35 ± 0.22 ^b^	21.38 ± 0.17 ^b^	1936.43 ± 192.70 ^a^	nd	nd	nd	30 [46]	Citrus, geranium [25]
		∑ Terpenes	55.77 ± 0.60 ^b^	47.55 ± 0.35 ^b^	2849.23 ± 230.50 ^a^		17.89 ± 0.58 ^b^			
1	1577	Isobutyric acid ^A^	747.25 ± 19.58 ^e^	2775.14 ± 171.63 ^c^	1029.00 ± 22.92 ^d^	15,624.12 ± 558.72 ^a^	4988.69 ± 437.36 ^b^	nd	2300 [47]	Fatty, rancid [30]
2	1638	Butanoic acid ^A^	173.67 ± 2.98 ^b^	180.24 ± 8.29 ^b^	nd	476.02 ± 10.62 ^a^	nd	nd	173 [47]	Cheese, rancid [30]
3	1682	Isovaleric acid ^A^	nd	519.01 ± 31.16 ^b^	nd	5579.41 ± 173.14 ^a^	nd	nd	33.4 [47]	Rancid [30]
4	2071	Octanoic acid ^A^	nd	107.13 ± 4.38 ^b^	nd	nd	1078.91 ± 40.80 ^a^	nd	500 [47]	Cheese, fatty, rancid [30]
5	2265	Decanoic acid ^D^	nd	76.66 ± 12.37 ^b^	nd	nd	1613.53 ± 137.53 ^a^	nd	1000 [47]	Fatty, rancid [30]
		∑ Fatty acids	920.91 ± 21.53 ^d^	3658.18 ± 207.93 ^c^	1029.00 ± 22.92 ^d^	21679.20 ± 429.27 ^a^	7681.12 ± 277.69 ^b^			
1	1010	4-Methyl-2-pentanone ^D^	234.09 ± 9.33 ^c^	237.13 ± 7.41 ^c^	380.54 ± 4.54 ^b^	211.62 ± 4.12 ^d^	402.13 ± 4.83 ^a^	nd	NF	NF
2	1130	3-Penten-2-one ^D^	nd	10.73 ± 0.36	nd	nd	nd	nd	NF	NF
3	1187	5-Methyl-2-hexanone ^D^	nd	nd	nd	nd	34.00 ± 1.42	0.60	NF	NF
4	1299	Acetoin ^A^	3480.97 ± 395.35 ^b^	25524.16 ± 3141.09 ^a^	nd	3911.90 ± 632.45 ^b^	tr	nd	150,000 [29,30]	Cream, butter [30]
5	1344	6-Methyl-5-hepten-2-one ^A^	1.72 ± 0.01 ^b^	2.25 ± 0.14 ^a^	nd	nd	nd	nd	NF	Fruity *
6	1402	Nonanal ^A^	13.48 ± 0.19 ^c^	13.85 ± 0.57 ^bc^	13.98 ± 0.21 ^bc^	14.20 ± 0.12 ^b^	15.12 ± 0.21 ^a^	13.10 ± 0.05	15 [23]	Green [28]
7	1396	2-Nonanone ^D^	nd	nd	nd	nd	194.55 ± 22.07	nd	NF	NF
8	1535	Benzaldehyde ^A^	20.81 ± 4.66 ^d^	85.19 ± 9.90 ^b^	59.36 ± 7.83 ^c^	22.40 ± 1.76 ^d^	133.71 ± 2.68 ^a^	10.54 ± 0.55	2000 [29,30]	Almond [31]
		∑ Carbonyl compounds	3751.07 ± 399.99 ^b^	25,873.31 ± 3145.56 ^c^	453.89 ± 11.52 ^d^	4160.11 ± 631.12 ^b^	779.51 ± 24.21 ^c^	24.23 ± 0.60		
1	1101	1-(1-ethoxyethoxy)-pentane ^D^	11.46 ± 0.53 ^c^	832.32 ± 85.35 ^a^	nd	215.96 ± 14.16 ^b^	288.58 ± 44.78 ^b^	nd	NF	NF
2	1643	*γ*-Butyrolactone ^D^	nd	26.95 ± 2.07	nd	nd	nd	nd	20,000 [29,30]	Caramel, sweet [29]
		∑ Other compounds	11.46 ± 0.53 ^c^	859.27 ± 87.25 ^a^		215.96 ± 14.16 ^b^	288.58 ± 44.78 ^b^			
		∑ All volatile aroma compounds	655,480.59 ± 50,073.53 ^b^	511,474.53 ± 28,041.48 ^c^	86,012.36 ± 2168.69 ^d^	1,123,301.80 ± 30,072.42 ^a^	1,086,723.08 ± 39,911.55 ^a^			

Values followed by different letters in a row are significantly different (*p* < 0.05) by Duncan test. “nd” means not detected; “tr = trace” means could not be quantified. The concentrations of volatile aroma compounds in the fermented medium of *Sac. vini* (BZL-28) and in the SGJ (uninoculated) were calculated by using calibration curve plotted in 1.0% (*v*/*v*) model wine solution. The concentrations of volatile aroma compounds in the fermented media of *M. guilliermondii* (AD-58), *Sat. diversa* (BZL-11), *W. anomalus* (DR-110), and *S. cerevisiae* (EC1118) were calculated by using calibration curve plotted in 8.0% (*v*/*v*) model wine solution. ^a^: Retention indices (RIs) of compounds on DB-Wax capillary column. ^b^: Compounds quantified methods. ^A^: calculated by internal standard-standard curve (plotted using corresponding standards); ^B^: calculated by internal standard-standard curve (plotted using the total areas of isomers); ^C^: estimated by internal standard-standard curve (compound with the most similar chemical structure); and ^D^: estimated by the ratio of areas between compounds and internal standard. ^c^: odor threshold: [23]: Odor threshold value was determined in a synthetic wine (10% *v*/*v* ethanol, 5 g/L tartaric acid, pH 3.2); [24]: Odor threshold values were determined in a synthetic wine (9.72 g/100 g water/ethanol, 5 g/L tartaric acid, pH 3.2); [29,30]: Odor threshold values were determined in a synthetic wine (10% *v*/*v* ethanol, pH 3.5); [46]: Odor threshold values were determined in water/ethanol (90/10, *w*/*w*); [47]: Odor threshold values were determined in a synthetic wine (11% *v*/*v* ethanol, 7 g/L glycerin, 5 g/L tartaric acid, pH 3.4); [48]: Odor threshold value was determined in a basic red wine; [49]: Odor threshold values were determined in water; [50]: Odor threshold value was determined in water; [51]: Odor threshold value was determined in a synthetic wine (12% *v*/*v* ethanol, 5 g/L tartaric acid, pH 3.5); and NF: Odor threshold values were not found in references. ^d^: odor description: * Described in the laboratory according to the odor of standards, Yunnan Agricultural University, China. NF: Odor descriptions were not found in references or no standards for described the odor in the laboratory.

### 3.3. PCA Analysis of Key Aroma Compounds

**Figure 2:**
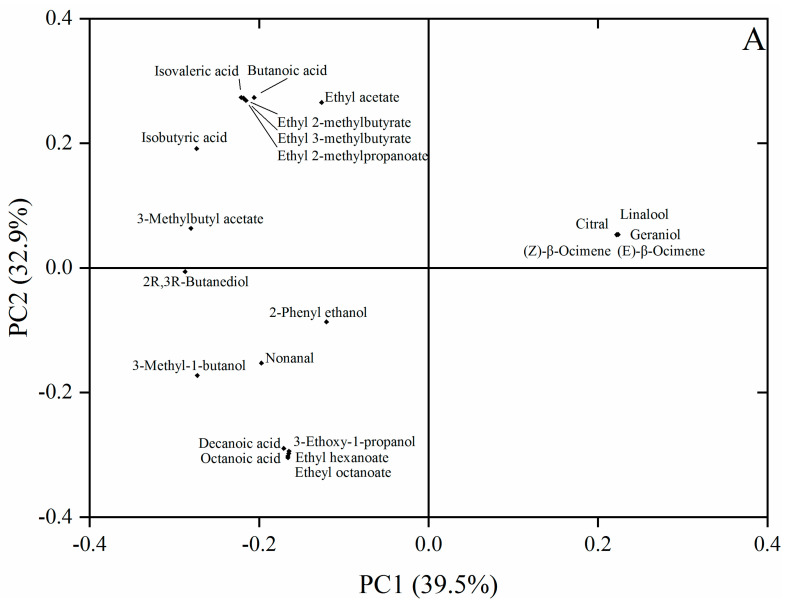

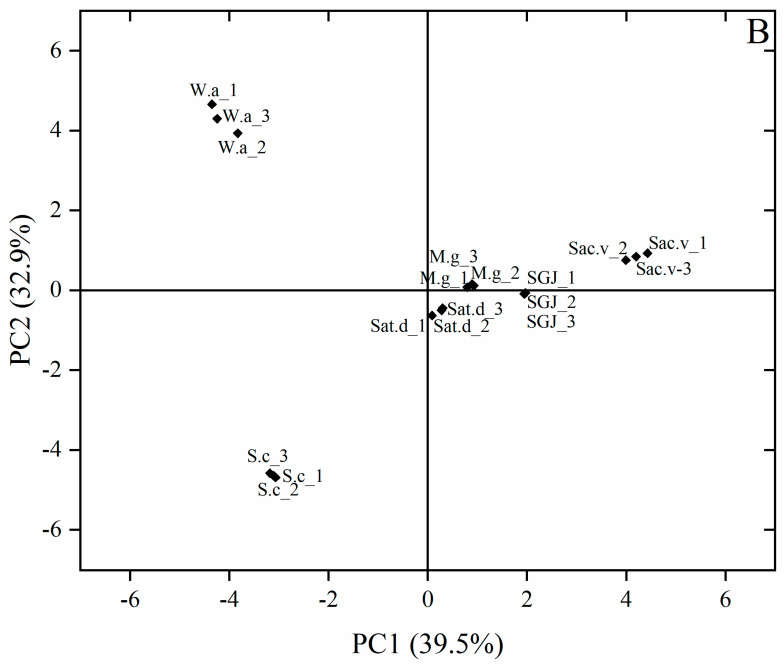
PCA analysis of key aroma compounds (**A**) and the distribution of five fermented media and SGJ (**B**) based on the first two principal components. M. g = *M. guilliermondii* (AD-58), Sat. d = *Sat. diversa* (BZL-11), Sac. v = *Sac. vini* (BZL-28), W. a = *W. anomalus* (DR-110), S. c = *S. cerevisiae* (EC1118), SGJ = Synthetic grape juice (uninoculated).

For the principal component analysis (PCA), the concentrations of 22 key aroma compounds were used to build up the data matrix and the distribution of the fermented media based on these key aroma compounds (Figure 2). A total of 72.4% variance was generated by the first two principal components, where 39.5% and 32.9% of the variance were explained by PC1 and PC2, respectively (Figure 2A). According to the proximity of these samples on the score plot, the fermented media of the four non-Saccharomyces yeast isolates and EC1118 were divided into four groups (Figure 2B). The fermented medium of EC1118 was located in the 3rd quadrant (Group 1) due to its greater ability to produce volatile aroma compounds such as 3-ethoxy-1-propanol, ethyl hexanoate, ethyl octanoate, octanoic acid, and decanoic acid. Group 2 was formed by the fermented medium of *W. anomalus* (DR-110), which showed greater production of ethyl acetate, ethyl 2-methylpropanoate, ethyl 2-methylbutyrate, ethyl 3-methylbutyrate, isobutyric acid, butanoic acid, and isovaleric acid. Group 3 was located in the 1st quadrant and was mainly formed by the fermented medium of *Sac. vini* (BZL-28), which showed greater production of (*Z*)-β-ocimene, (*E*)-β-ocimene, linalool, citral, and geraniol. The uninoculated SGJ and the fermented media of *M. guilliermondii* (AD-58) and *Sat. diversa* (BZL-11) were closely grouped together due to their low concentrations of most of the volatile compounds. 

### 3.4. Aroma Profile Analysis of Resultant Fermented Media

**Figure 3:**
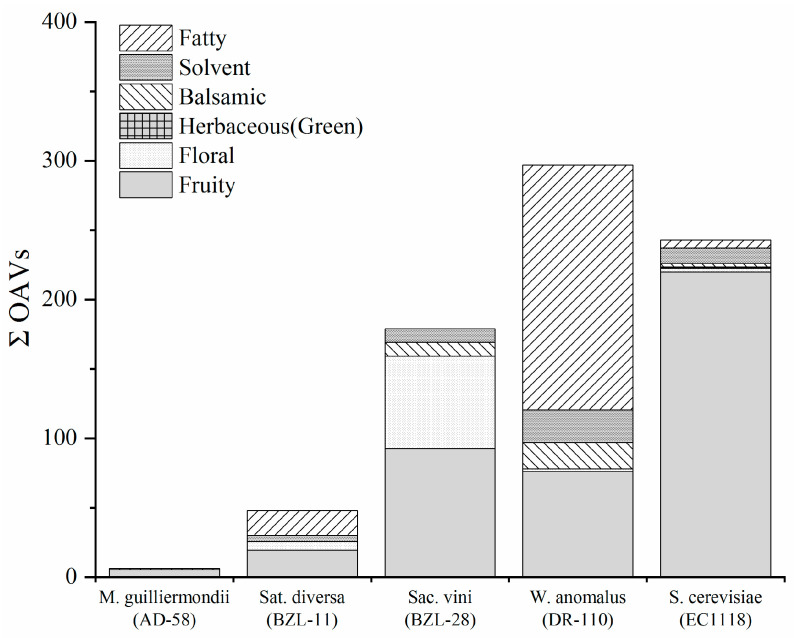
Sum of OAVs of different aroma series in five fermented media.

To understand the composition of the different aroma series (categories) in each of the individual fermented media, the total OAV (ΣOAVs) was calculated by summing up the OAVs of the individual compounds (with OAVs > 1.0) belonging to each aroma series (Table 3). As shown in Figure 3, the fermented media of the four non-*Saccharomyces* yeast isolates exhibited completely different aroma profiles when compared with the fermented medium of EC1118, which was primarily characterized by a fruity aroma. The fermented medium of *W. anomalus* (DR-110) was largely characterized by a fatty aroma due to its high production of short-chain fatty acids and a fruity aroma due to its great production of esters. The fermented medium of *Sac. vini* (BZL-28) was largely characterized by fruity (citrus-like) and floral aromas, which were related to its high concentration of monoterpenes. The fermented medium of *Sat. diversa* (BZL-11) was also characterized by fruity and fatty aromas, but with much lower ΣOAVs compared with the medium that was fermented by *W. anomalus* (DR-110). No distinctive aroma was observed in the fermented medium of *M. guilliermondii* (AD-58) due to the very low OAVs of most of the volatiles (Table 3).

**Table 3:** OAVs of key aroma compounds in five fermented media and SGJ.

No.	Compounds	*M. guilliermondii*(AD-58)	*Sat. diversa* (BZL-11)	*Sac. vini* (BZL-28)	*W. anomalus* (DR-110)	*S. cerevisiae* (EC1118)	SGJ (Uninoculated)	Aroma Series
1	3-Methyl-1-butanol	0.72 ± 0.02c	4.31 ± 0.27b	0.19 ± 0.02d	4.36 ± 0.07b	8.34 ± 0.44a	-	Solvent [30]
2	3-Ethoxy-1-propanol	-	-	-	-	5.29 ± 0.20	-	Fruity [30]
3	2-Phenylethanol	0.37 ± 0.01d	6.35 ± 0.30a	0.02 ± 0.01e	1.74 ± 0.04c	2.67 ± 0.04b	-	Floral [30]
4	2R,3R-Butanediol	3.58 ± 0.30c	1.55 ± 0.08d		4.15 ± 0.20a	3.84 ± 0.16b	-	Fruity [30]
5	Ethyl acetate	0.89 ± 0.04d	0.23 ± 0.03e	9.88 ± 0.39b	19.07 ± 0.65a	2.58 ± 0.12c	-	Fruity, Balsamic, Solvent [30]
6	3-Methylbutyl acetate	1.70 ± 0.12d	0.47 ± 0.12e	3.54 ± 0.93c	16.90 ± 0.86a	12.54 ± 0.76b	-	Fruity [30]
7	Ethyl 2-methylpropanoate	-	-	-	25.43 ± 4.65	-	-	Fruity [30]
8	Ethyl 2-methylbutyrate	-	-	-	7.03 ± 0.68	-	-	Fruity [26]
9	Ethyl 3-methylbutyrate	-	-	-	1.45 ± 0.39	-	-	Fruity [26]
10	Ethyl hexanoate	-	2.41 ± 0.10b	-	2.20 ± 0.07b	87.05 ± 9.92a	-	Fruity [30]
11	Etheyl octanoate	-	15.45 ± 2.17b	-	-	108.51 ± 4.18a	-	Fruity [30]
12	(*Z*)-β-Ocimene	-	-	3.27 ± 0.31	-	-	-	Fruity [27]
13	(*E*)-β-Ocimene	-	-	5.17 ± 0.55	-	-	-	Fruity [27]
14	Linalool	-	-	1.92 ± 0.14	-	-	-	Fruity, Floral [30]
15	Citral	-	-	4.41 ± 0.33	-	-	-	Fruity *
16	Geraniol	0.65 ± 0.01b	0.71 ± 0.01b	64.55 ± 6.42a	-	-	-	Fruity, Floral [25]
17	Isobutyric acid	0.32 ± 0.01d	1.21 ± 0.07c	0.45 ± 0.01d	6.79 ± 0.24a	2.17 ± 0.19b	-	Fatty [30]
18	Butanoic acid	1.00 ± 0.02b	1.04 ± 0.05b	-	2.75 ± 0.06a	-	-	Fatty [30]
19	Isovaleric acid	-	15.54 ± 0.93b	-	167.05 ± 5.18a	-	-	Fatty [30]
20	Octanoic acid	-	0.21 ± 0.01b	-	-	2.16 ± 0.08a	-	Fatty [30]
21	Decanoic acid	-	0.08 ± 0.01b	-	-	1.61 ± 0.14a	-	Fatty [30]
22	Nonanal	0.90 ± 0.01cd	0.92 ± 0.04bc	0.93 ± 0.01bc	0.95 ± 0.01b	1.01 ± 0.01a	0.87 ± 0.00d	Herbaceous (Green) [28]

Values followed by different letters in a row are significantly different (*p* < 0.05) by Duncan test. *: Aroma series were classified in the laboratory according to the odor of standards, Yunnan Agricultural University, China.

## 4. Conclusions

This study described the fermentation performances and aroma compositions of four indigenous non-*Saccharomyces* yeast isolates, *Meyerozyma guilliermondii* (AD-58), *Saccharomycopsis vini* (BZL-28), *Saturnispora diversa* (BZL-11), and *Wickerhamomyces anomalus* (DR-110) in the fermentation of synthetic grape juice. *Sat. diversa* (BZL-11) and *Sac*. *vini* (BZL-28) showed the highest and the lowest fermentation capacity, respectively, whereas *M*. *guilliermondii* (AD-58) and *W*. *anomalus* (DR-110) exhibited an intermediate fermentative capacity. These four indigenous non-*Saccharomyces* yeast isolates showed lower conversion rates of sugar to alcohol and higher yields of volatile acidity. *W. anomalus* (DR-110) had a greater ability to produce numerous esters and short-chain fatty acids, which contributed to the fruity and fatty aromas in its fermented medium. *Sac. vini* (BZL-28) exhibited a great capacity in the formation of monoterpenes, especially (*Z*)-β-ocimene, (*E*)-β-ocimene, linalool, citral, and geraniol, which can enhance the fruity (citrus-like) and floral aromas in the resultant fermented medium. Although *M. guilliermondii* (AD-58) and *Sat. diversa* (BZL-11) showed some potential in the production of 2,3-butanediol and 2-phenylethanol, respectively, the concentrations of most of the other volatiles that were produced by these two isolates were much lower than their sensory thresholds. Therefore, they may have little impact on the aroma profiles of the resultant fermented media. Our study provides more insights into the four indigenous non-*Saccharomyces* yeast isolates that were obtained from the Shangri-La wine region in China. These non-*Saccharomyces* yeast isolates may play an important role in shaping the regional characteristics of the wines that are produced from this region. Further studies on these non-*Saccharomyces* yeast isolates in the fermentation of local grapes at an industrial scale will provide us with more valuable information for their application in producing wines with more regional characteristics.

## Figures and Tables

**Table 1:** Oenological parameter analysis of five fermented media and SGJ (mean ± SD).

	*M. guilliermondii* (AD-58)	*Sat. diversa* (BZL-11)	*Sac. vini* (BZL-28)	*W. anomalus* (DR-110)	*S. cerevisiae* (EC1118)	SGJ (Uninoculated)
Reducing (residual) sugar (g/L)	88.06 ± 2.19b	16.35 ± 1.12d	167.28 ± 1.44a	57.73 ± 1.53c	0.75 ± 0.01e	173.66 ± 0.87
Alcohol content (% *v*/*v*)	4.40 ± 0.08d	8.41 ± 0.02b	0.06 ± 0.02e	5.39 ± 0.03c	9.54 ± 0.05a	0.01 ± 0.01
pH (20 °C)	3.42 ± 0.01b	3.31 ± 0.02c	3.50 ± 0.02a	3.32 ± 0.01c	3.26 ± 0.02d	3.50 ± 0.02
Total acidity (g/L as tartatic acid)	5.86 ± 0.04a	5.19 ± 0.06b	3.28 ± 0.04d	5.81 ± 0.06a	4.93 ± 0.04c	2.69 ± 0.02
Volatile acidity (g/L as accetic acid)	1.96 ± 0.02a	0.79 ± 0.02b	0.44 ± 0.00c	1.97 ± 0.06a	0.25 ± 0.02d	0.06 ± 0.00
Reducing sugar consumption (g/L)	85.59 ± 2.19d	157.31 ± 1.12b	6.37 ± 1.44e	115.93 ± 1.53c	172.91 ± 0.01a	-
Sugars used for 1% ethanol production (g)	19.45	18.70	106.17	21.51	18.12	-

Values followed by different letters in a row are significantly different (*p* < 0.05) by Duncan test.

## Data Availability

All data analyzed or generated during this study are available within the manuscript and can be requested from the corresponding author.

## Supplementary Materials

The following are available online at https://www.mdpi.com/article/10.3390/jof8020146/s1, Figure S1: The colony morphologies of four non-Saccharomyces yeast isolates on YPD (A, B, C, D) and WL (a, b, c, d) agar, Figure S2: Phylogenetic tree of four non-Saccharomyces yeast isolates based on the sequence analysis of the 26S rRNA D1/D2 region using the maximum-likelihood method. The scale bar shows 0.05, Bootstrap support values were estimated based on 1000 replicates. Figure S3: Phylogenetic tree of four non-Saccharomyces yeast isolates based on the sequence analysis of the 5.8S rRNA ITS region using the maximum-likelihood method. The scale bar shows 0.05, Bootstrap support values were estimated based on 1000 replicates. Table S1: Detailed information of 26S rRNA D1/D2 region and 5.8S rRNA ITS region of four non-Saccharomyces yeast isolates, Table S2: Identification methods, quantitative standards and calibration curves of volatile aroma compounds.
