# Supplementary material for: Evaluation of Four Indigenous Non-Saccharomyces Yeasts Isolated from the Shangri-La Wine Region (China) for Their Fermentation Performances and Aroma Compositions in Synthetic Grape Juice Fermentation
Source: J Fungi (Basel). 2022 Jan 30;8(2):146. doi: 10.3390/jof8020146 (PMC8879568; doi:10.3390/jof8020146)
Supplement: Supplementary file 1 [file jof-08-00146-s001.zip › jof-1564787-supplementary.pdf]

Supplementary data

Table S1 Detailed information of 26S rRNA D1/D2 region and 5.8S rRNA ITS region of four non-*Saccharomyces* yeast isolates.

Species (Strain)	26S rRNA D1/D2 region		5.8S rRNA ITS region	
	Fragment Size (bp)	Gen Bank accession numbers	Fragment Size (bp)	Gen Bank accession numbers
Meyerozyma guilliermondii (AD-58)	586	MN371925	578	MN371912
Saccharomycopsis vini (BZL-28)	580	MN371928	575	MN371909
Saturnispora diversa (BZL-11)	546	MN371926	377	MN371911
Wickerhamomyces anomalus (DR-110)	577	MN371940	591	MN371897

Table S2 Identification methods, quantitative standards and calibration curves of volatile aroma compounds

No.	Compound	CAS	RI[1]	Identification[2]	Quantitative standards	Calibration curves[3]	R^2
	**Higher alcohols**						
1	1-Propanol	71-23-8	1036	A	1-Propanol	y=78880.76x+283.05	0.992
						*y=101171.67x-291.63	*0.999
2	2-Methyl-1-propanol	78-83-1	1085	A	2-Methyl-1-propanol	y=12472.11x-2026.42	0.998
						*y=27221.21x-44.37	*0.992
3	Butanol	71-36-3	1142	A	Butanol	y=17005.28x-243.71	0.997
4	3-Methyl-1-butanol	123-51-3	1213	A	3-Methyl-1-butanol	y=5854.17x-66.48	0.995
						*y=5605.10x-37.07	*0.996
5	3-Methyl-3-buten-1-ol	763-32-6	1254	A	3-Methyl-3-buten-1-ol	y=3516.67x+4.70	0.998
6	3-Methyl-1-pentanol	589-35-5	1333	A	3-Methyl-1-pentanol	y=1184.99x+0.53	0.999
7	3-Ethoxy-1-propanol	111-35-3	1386	A	3-Ethoxy-1-propanol	y=99612.46x-3156.06	0.996
8	2-Ethyl-1-hexanol	104-76-7	1498	A	2-Ethyl-1-hexanol	y=74.05x+1.75	0.999
						*y=122.97x-2.14	*0.998
9	2-Nonanol	628-99-9	1527	B	Nonanol	y=50.00x+6.81	0.996

						*y=105.15x+5.79	*0.999
10	Octanol	111-87-5	1566	A	Octanol	y=75.52x+4.22	0.999
						*y=140.13x+0.81	*0.999
11	Nonanol	143-08-8	1669	A	Nonanol	y=50.00x+6.81	0.996
						*y=105.15x+5.79	*0.999
12	3-Methylthio-1-propanol	505-10-2	1730	A	3-Methylthio-1-propanol	y=18566.74x+2.51	0.998
13	Benzyl alcohol	100-51-6	1891	A	Benzyl alcohol	y=5299.90x+43.48	0.999
						*y=5683.08x+38.55	*0.999
14	2-Phenylethanol	60-12-8	1928	A	2-Phenylethanol	y=4344.55x-164.68	0.986
						*y=3723.87x+50.50	*0.999
	**Polyols**						
1	2R,3R-Butanediol	24347-58-8	1549	B	2R,3S-Butanediol	y=1172078.80x-90.76	0.986
2	2R,3S-Butanediol	5341-95-7	1585	A	2R,3S-Butanediol	y=1172078.80x-90.76	0.986
	**Acetate esters**						
1	Ethyl acetate	141-78-6	935	A	Ethyl acetate	y=7702.52x-63.10	0.997
						*y=9816.85x-48.96	*0.999
2	Propyl acetate	109-60-4	985	A	Propyl acetate	y=1280.09x-35.15	0.998
						*y=3577.12x-54.12	*0.999
3	3-Methylbutyl acetate	123-92-2	1118	A	3-Methylbutyl acetate	y=172.07x-1.80	0.999
						*y=602.56x-8.94	*0.999
4	Geranyl acetate	105-87-3	1764	A	Geranyl acetate	*y=43806.71x+5.01	*0.998
5	Phenylethyl acetate	103-45-7	1829	A	Phenylethyl acetate	y=247.55x+14.40	0.998
	**Fatty acid ethyl esters**						
1	Ethyl propaonate	105-37-3	974	A	Ethyl propaonate	y=2402.71x-99.01	0.999
						*y=6388.89x-169.66	*0.998
2	Ethyl 2-methylpropanoate	97-62-1	979	A	Ethyl 2-methylpropanoate	y=400.21x+41.51	0.995

3	Ethyl 2-methylbutyrate	7452-79-1	1048	A	Ethyl 2-methylbutyrate	y=290.22x-1.25	0.999
4	Ethyl 3-methylbutyrate	108-64-5	1063	A	Ethyl 3-methylbutyrate	y=256.97x-0.14	0.999
5	Ethyl hexanoate	123-66-0	1239	A	Ethyl hexanoate	y=81.85x+7.95	0.999
6	Ethyl octanoate	106-32-1	1444	A	Ethyl octanoate	y=80.31x+17.55	0.997
7	Ethyl decanoate	110-38-3	1649	A	Ethyl decanoate	y=191.14x+22.39	0.998
8	Ethyl phenylacetate	101-97-3	1797	A	Ethyl phenylacetate	y=192.17x+16.32	0.998
9	Ethyl dodecanoate	106-33-2	1854	A	Ethyl dodecanoate	y=171.71x+18.41	0.998
10	Ethyl tetradecanoate	124-06-1	2053	B	-	**y=2030x	
11	Ethyl hexadecanoate	628-97-7	2243	A	Ethyl hexadecanoate	y=1294.7x+5.97	0.995
	**other esters**						
1	2-Methylpropyl 2-methylbutanoate	2445-67-2	1182	B	-	**y=2030x	
2	3-Methylbutyl propionate	105-68-0	1196	A	3-Methylbutyl propionate	y=104.62x+6.24	0.998
3	3-Methylbutyl 2-methylpropanoate	2050-01-3	1202	B	-	**y=2030x	
4	3-Methylbutyl 2-methylbutanoate	27625-35-0	1285	B	-	**y=2030x	
5	2-Methylbutyl 2-methylbutanoate	2445-78-5	1288	B	-	**y=2030x	
	**Terpenes**						
1	*β*-Myrcene	123-35-3	1163	A	*β*-Myrcene	*y=2090.89x+14.61	*0.998
2	D-Limonene	5989-27-5	1207	A	D-Limonene	*y=1884.95x+3.35	*0.995
3	(*Z*)-*β*-Ocimene	3338-55-4	1238	A	(*Z*) & (*E*)-*β*-Ocimene	*y=4531.22x+6.63	*0.993
4	(*E*)-*β*-Ocimene	3779-61-1	1256	A	(*Z*) & (*E*)-*β*-Ocimene	*y=4531.22x+6.63	*0.993
5	Linalool	78-70-6	1554	A	Linalool	*y=161.76x+9.56	*0.999
6	*α*-Terpineol	98-55-5	1711	A	*α*-Terpineol	y=259.15x+8.36	0.999
						*y=194.29x+8.19	*0.999
7	Citral	5392-40-5	1745	A	Citral	*y=3273.54x+44.80	*0.997
8	Citronellol	106-22-9	1774	A	Citronellol	y=119.45x+8.68	0.998
						*y=190.91x-4.63	*0.998

9	Nerol	106-25-2	1810	A	Nerol	y=278.03x+14.72	0.999
						*y=444.63x+12.58	*0.999
10	Geraniol	106-24-1	1858	A	Geraniol	y=353.36x+17.51	0.999
						*y=398.22x+15.43	*0.999
	**Fatty acids**						
1	Isobutyric acid	79-31-2	1577	A	Isobutyric acid	y=23353.68x+142.05	0.993
						*y=18813.94x+40.49	*0.997
2	Butanoic acid	107-92-6	1638	A	Butanoic acid	y=20068.70x+39.73	0.998
3	Isovaleric acid	503-74-2	1682	A	Isovaleric acid	y=6672.94x+4.61	0.991
4	Octanoic acid	124-07-2	2071	A	Octanoic acid	y=751.96x+75.23	0.997
5	Decanoicacid	334-48-5	2265	B	-	**y=2030x	
	**Carbonyl compounds**						
1	4-Methyl-2-pentanone	108-10-1	1010	B	-	**y=2030x	
2	3-Penten-2-one	625-33-2	1130	B	-	**y=2030x	
3	5-Methyl-2-hexanone	110-12-3	1187	B	-	**y=2030x	
4	Acetoin	513-86-0	1299	A	Acetoin	y=127541.70x-203.01	0.993
5	6-Methyl-5-hepten-2-one	110-93-0	1344	A	6-Methyl-5-hepten-2-one	y=126.68x+1.39	0.999
7	2-Nonanone		1396	B	-	**y=2030x	
6	Nonanal	124-19-6	1402	A	Nonanal	y=66.38x+12.98	0.998
						*y=522.66x+14.02	*0.999
8	Benzaldehyde	100-52-7	1535	A	Benzaldehyde	y=338.47x+10.09	0.999
						*y=411.90x+7.36	*0.999
	**Other compounds**						
1	1-(1-ethoxyethoxy)-pentane	13442-89-2	1101	B	-	**y=2030x	
2	γ-Butyrolactone	96-48-0	1643	B	-	**y=2030x	

1. Retention indices (RIs) were calculated on DB-Wax capillary column.

2. Identification of volatile aroma compounds: A, mass spectrum and RI agreed with standards; B, mass spectrum and RI agreed with NIST 14 MS database and literature data.

3. In calibration curves, “y” means the concentration of volatile aroma compound (μg/L); “x” means the area ratio of volatile aroma compound to internal standard (4-methyl-2-pentanol). “*” means the calibration curve of these compounds were plotted in 1.0 % (v/v) model wine solution. “**” means the concentration of these compounds was expressed as relative amount compared to internal standard.

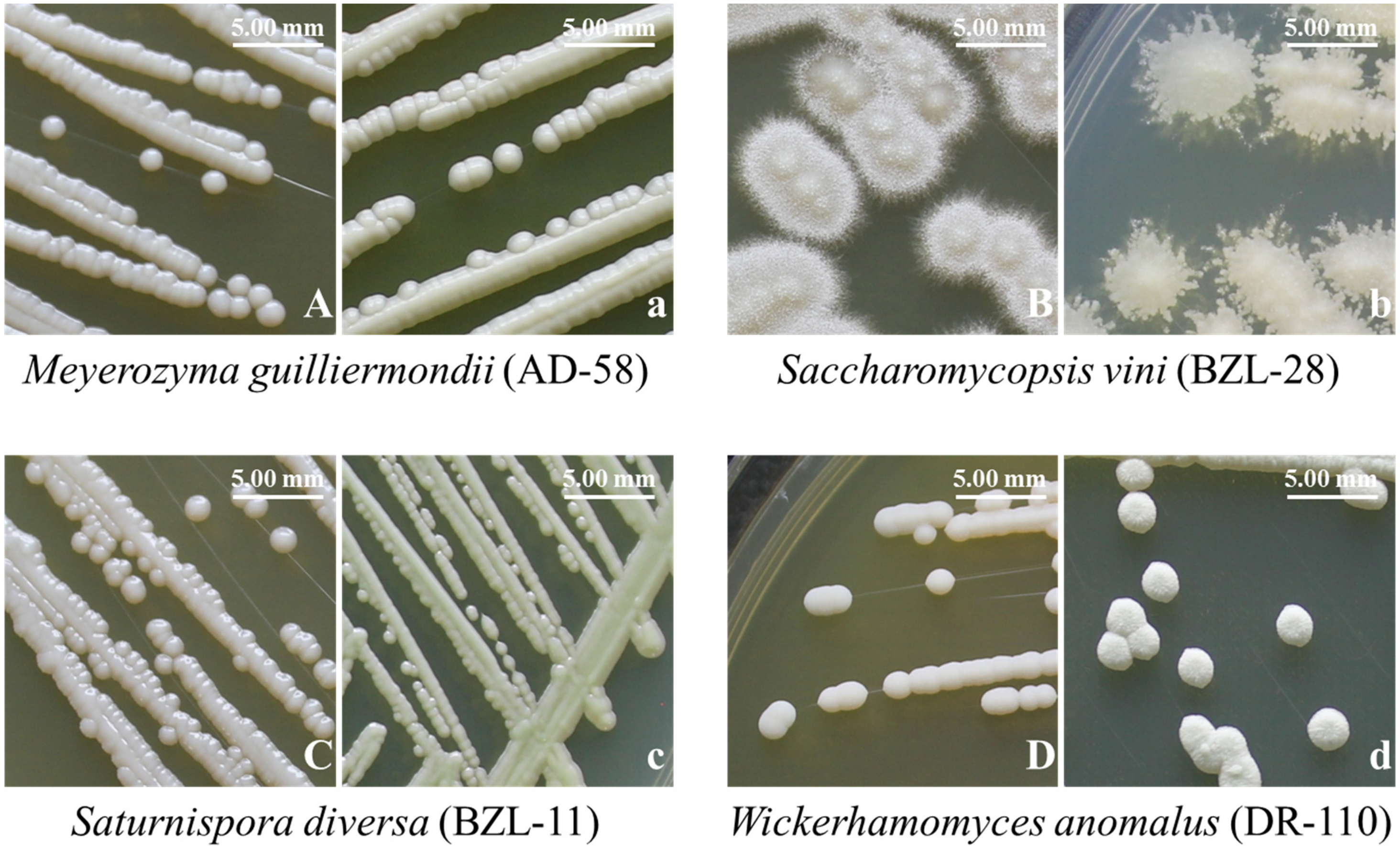

Figure S1 The colony morphologies of four non-Saccharomyces yeast isolates on YPD (A, B, C, D) and WL (a, b, c, d) agar.

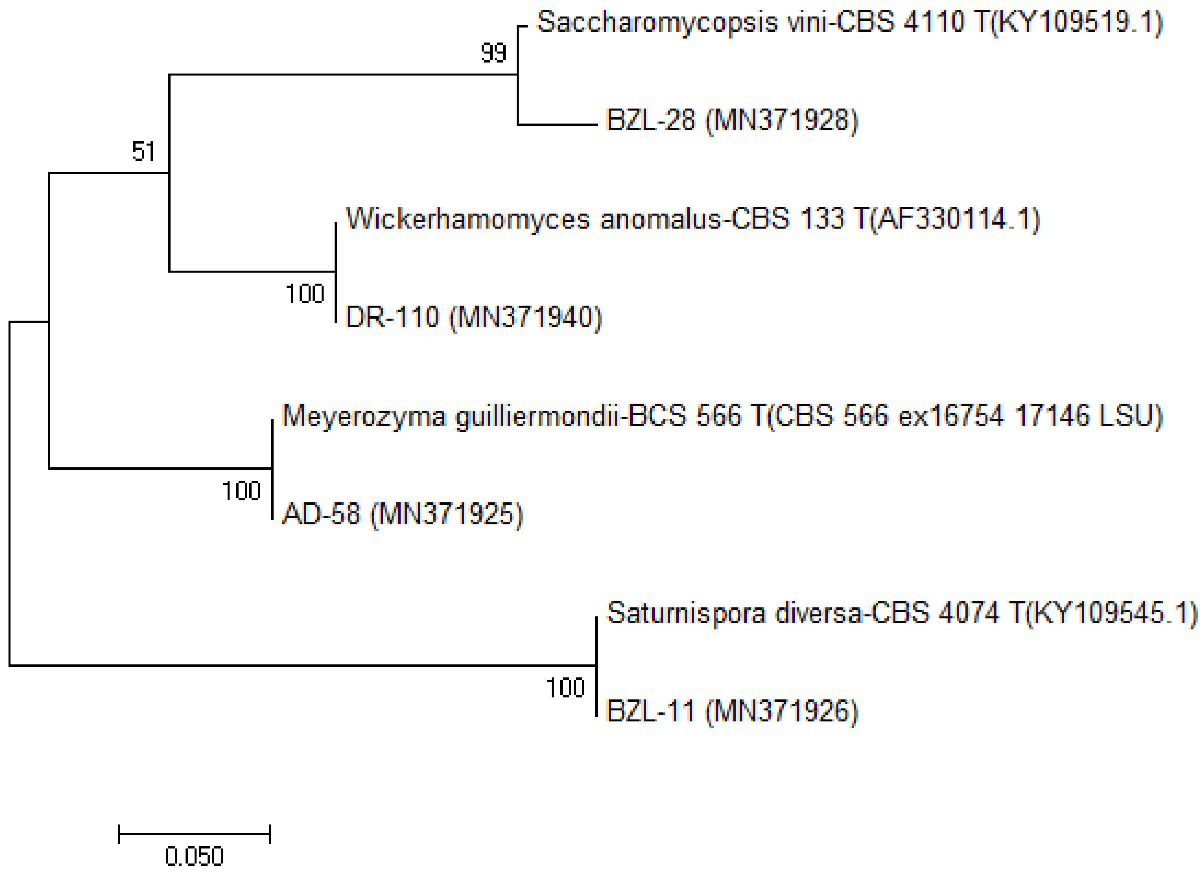

Figure S2 Phylogenetic tree of four non-*Saccharomyces* yeast isolates based on the sequence analysis of the 26S rRNA D1/D2 region using the maximum-likelihood method. The scale bar shows 0.05, Bootstrap support values were estimated based on 1000 replicates.

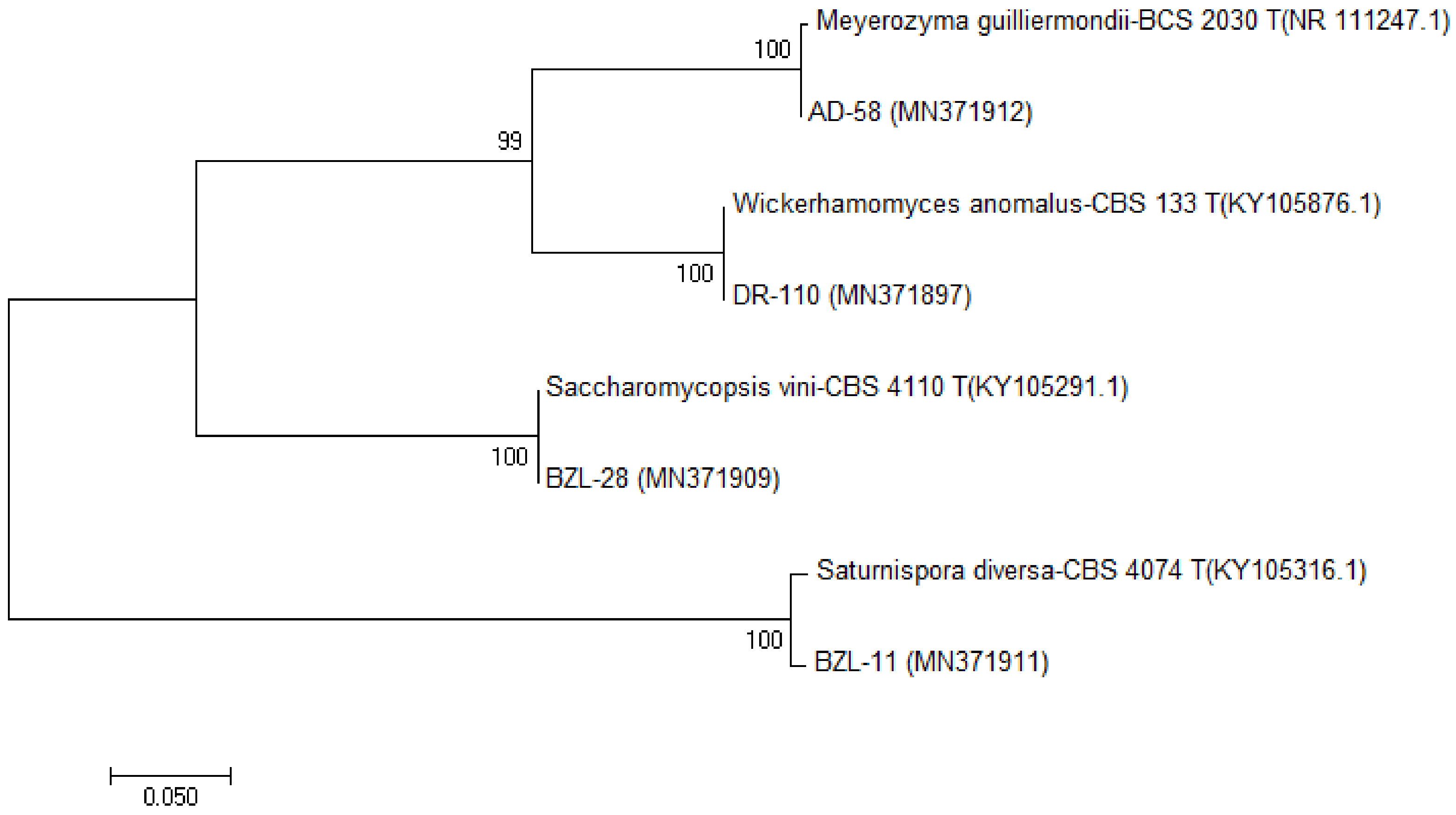

Figure S3 Phylogenetic tree of four non-*Saccharomyces* yeast isolates based on the sequence analysis of the 5.8S rRNA ITS region using the maximum-likelihood method. The scale bar shows 0.05, Bootstrap support values were estimated based on 1000 replicates.
